# An In Vitro Strategy to Evaluate Ketoprofen Phototoxicity at the Molecular and Cellular Levels

**DOI:** 10.3390/ijms252312647

**Published:** 2024-11-25

**Authors:** Klaudia Banach, Justyna Kowalska, Mateusz Maszczyk, Zuzanna Rzepka, Jakub Rok, Dorota Wrześniok

**Affiliations:** Department of Pharmaceutical Chemistry, Faculty of Pharmaceutical Sciences in Sosnowiec, Medical University of Silesia, 40-055 Katowice, Poland; jkowalska@sum.edu.pl (J.K.); matmaszczyk@proton.me (M.M.); zrzepka@sum.edu.pl (Z.R.); jrok@sum.edu.pl (J.R.); dwrzesniok@sum.edu.pl (D.W.)

**Keywords:** ketoprofen, phototoxic dermatitis, epidermal cells, adverse effects

## Abstract

Phototoxicity is a significant problem that occurs in a large part of the population and is often caused by commonly used pharmaceuticals, including over-the-counter drugs. Therefore, testing drugs with photosensitizing potential is very important. The aim of this study is to analyze the cytotoxicity and phototoxicity of ketoprofen towards human melanocytes and fibroblasts in three different treatment schemes in order to optimize the study. Cytometric tests (studies of viability, proliferation, intracellular thiol levels, mitochondrial potential, cell cycle, and DNA fragmentation), Western blot analysis (cytochrome c and p44/p42 protein levels), and confocal microscopy imaging were performed to assess the impact of the developed treatments on skin cells. Research on experimental schemes may help reduce or eliminate the risk of phototoxic reactions. In the case of ketoprofen, we found that the strongest phototoxic potential was exhibited in the treatment where the drug was present in the solution during the irradiation of cells, both pigmented and non-pigmented cells. These results indicate that the greatest risk of photosensitivity reactions related to ketoprofen occurs after direct contact with the drug and UV exposure.

## 1. Introduction

Skin forms a physical, chemical, and immunological barrier, which enables effective protection of the internal environment of the body from harmful external factors. In addition to this primary function, it is also involved in maintaining homeostasis [1,2].

An important element of the epidermis is melanocytes, which have the ability to phagocytose and express antigens of the major histocompatibility system. Their other function is to produce melanin, a pigment that protects against the harmful effects of ultraviolet (UV) radiation [3,4]. The degree of sensitivity of the skin to the damaging effects of UV radiation mainly depends on the amount and type of melanin [4,5]. Eumelanin is a pigment characterized by its brown to black color, and it is synthesized in larger quantities in people with dark skin phototypes. The mechanism of its photoprotective effect is absorbing UV radiation and neutralizing reactive oxygen species (ROS) and free radicals. Red–yellow pheomelanin is less effective at blocking radiation. This dye is susceptible to photodegradation, which results in generating superoxide anions and hydrogen peroxide that can cause mutations in melanocytes and other cells [6,7,8,9].

UVA radiation, a type of UV of 315–400 nm wavelength, penetrates deeply, reaching the dermis. It causes most of the photosensitivity reactions and has a strong pigment-forming effect. Its harmful effects are associated with the generation of reactive species, which leads to the oxidation of cell membrane lipids and direct cellular damage. It can lead to development of skin cancer [10,11]. In addition, the radiation induces photoaging of the skin through the accumulation of abnormal elastin fibers and the simultaneous degradation of collagen fibers and other components of the intercellular matrix [10,11,12,13,14].

The use of drugs and other substances, both orally and topically, can cause the formation of very painful and difficult-to-treat skin lesions, such as photosensitivity reactions. This includes phototoxicity and photoallergy. Drug-induced photosensitivity reactions are common, but rarely reported due to misdiagnosis or no clinical recognition, as most cases may go undetected or be mistaken for mild sunburn [15,16,17,18].

A critical aspect of photosensitivity reactions is the fact that they can be triggered by commonly used drugs, and even those available without a prescription. Ketoprofen is one of the most common pharmaceuticals that can cause skin rash in sun-exposed areas. It is used in the treatment of musculoskeletal and joint disorders, osteoarthritis, ankylosing spondylitis, and rheumatoid arthritis, as well as in periarticular disorders such as tendinitis and in mild to moderate pain. The induction of a light hypersensitivity reaction in the case of ketoprofen may be due to its chemical structure, which leads to photosensitivity reactions. This drug can induce a phototoxic reaction through the production of singlet oxygen and free radicals, leading to skin lesions. It is considered that the benzophenone moiety in the ketoprofen structure plays a major role in its ability to act as a photosensitizer [19,20,21].

Ketoprofen phototoxicity is an important medical issue. The scientific literature describes numerous cases of patients who have experienced photosensitivity reactions associated with the use of this drug. In case reports, patients using ketoprofen for the treatment of pain or inflammation have been reported to develop severe skin reactions after brief exposure to sunlight. These skin lesions manifested as very painful, hard-to-heal conditions such as erythema, blisters, and even second-degree burns. Additionally, post-inflammatory hyperpigmentation or scarring may persist on the skin after these reactions [22,23]. The available scientific literature describes the results of in vitro studies demonstrating the significant effects of ketoprofen and radiation on the morphology and function of keratinocytes and Langerhans cells [24]. It has been reported that ketoprofen under UVA irradiation significantly affects the viability and function of cancer cells, which can be used to support cancer therapy, including breast cancer and melanoma [25,26]. However, these studies still do not fully describe the molecular aspects of ketoprofen phototoxicity. Expanding knowledge on this subject could help in the development of appropriate clinical recommendations.

Since the pathomechanism of phototoxicity is complex and involves different skin cell types, we decided to analyze the cytotoxic and phototoxic activity of ketoprofen towards human melanocytes and fibroblasts. To determine the optimal conditions, three different treatment schemes were performed. In addition, the use of different types of cells, that is, pigmented and non-pigmented, allowed us to compare the reaction of the cells to the phototoxic effects of the drug.

## 2. Results and Discussion

### 2.1. The Influence of Ketoprofen and UVA on the Cell Viability and Proliferation of Normal Skin Cells

Cell viability and proliferation are essential parameters of the cell culture and often form the basis for further research. In order to examine them, a method that utilizes two dyes was used, which were acridine orange—AO (stains both living and dead cells)—and DAPI (stains dead cells) [27].

**Figure 1:**
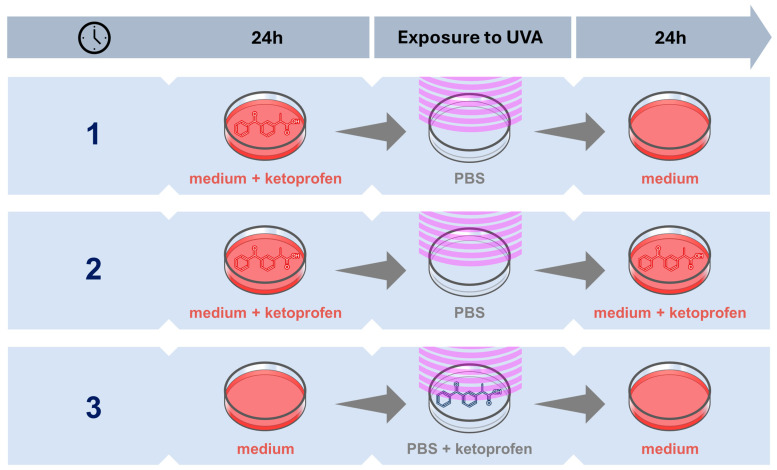
Graphical representation of the developed treatment schemes (1–3).

Under UVA radiation, ketoprofen undergoes photodegradation, resulting in the formation of free radicals, which are responsible for skin cell damage. Taking this into account, we decided to analyze the phototoxic effect of ketoprofen in three treatment schemes on pigmented cells—melanocytes (HEMn-LP)—and non-pigmented cells—dermal fibroblasts (HDFs). These include cells cultured in a medium with ketoprofen prior to UVA irradiation (Treatment Scheme 1), cells cultured in a medium with ketoprofen before and after being exposed to UVA (Treatment Scheme 2), and cells irradiated with UVA and treated with ketoprofen at the same time (Treatment Scheme 3)—Figure 1.

**Figure 2:**
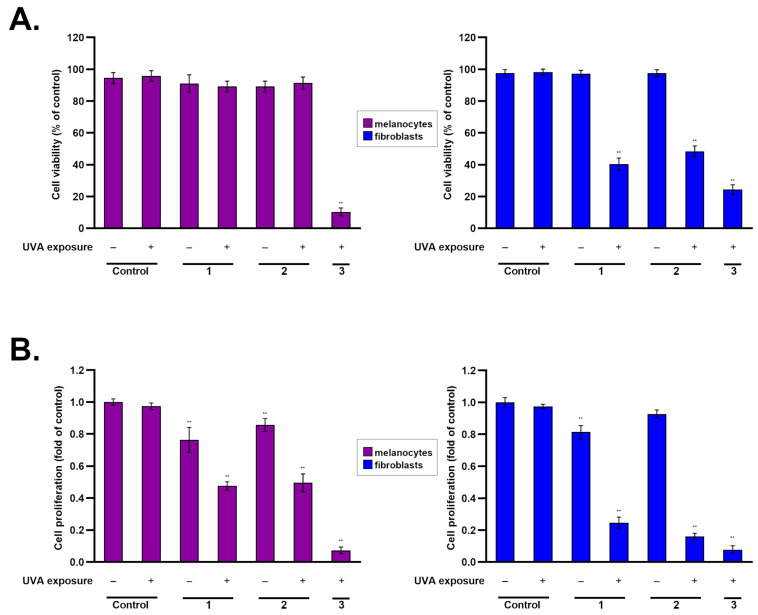
Effects of ketoprofen and UVA (in three treatment schemes, 1–3) on viability (**A**) and proliferation (**B**) of melanocytes and fibroblasts. Bar graphs present mean values ± SD; ** *p* < 0.001 vs. control.

The first examined parameter of the studied schemes was cell viability. A culture of normal human cells was assessed after being exposed to ketoprofen and UVA radiation. The analysis was based on the evaluation of living cells in relation to the whole population. The research conducted on melanocytes showed a significant decrease in viability only in one scheme—the group exposed simultaneously to UVA and the drug. In turn, in fibroblasts, a significant reduction in their viability was observed in all treatments irradiated with UVA (decrease by approximately: Treatment Scheme 1—60%, Treatment Scheme 2—55%, Treatment Scheme 3—80% in relation to the control)—Figure 2A.

Another examined parameter was cell proliferation, which was determined using the coefficient related to the control. In melanocytes and fibroblasts, ketoprofen without irradiation caused a reduction in proliferation. However, this effect was significantly deepened after the exposure of cells to UVA radiation in all research models. The greatest antiproliferative effect against both melanocytes and fibroblasts was found in cells treated with Treatment Scheme 3 with UVA, where the ratio was about 0.07 for melanocytes and 0.08 for fibroblasts—Figure 2B.

The assessment of viability and proliferation of the cells showed that the impact of ketoprofen in combination with irradiation was stronger in fibroblasts than in melanocytes. This may be due to the protective role of melanin localized in melanocytes. The main function of this group of biopolymers is to protect cells from the harmful effects of UV radiation and to eliminate free radicals, primarily ROS [28,29,30]. Eumelanin is responsible for removing free radicals by reducing superoxide anions to hydrogen peroxide. Moreover, melanins can also form complexes with drugs, thus influencing their therapeutic effectiveness and toxicity [31,32,33]. These presumptions are in line with the study conducted on dark-pigmented melanocytes (HEMn-DP), where the effect of ketoprofen and UVA on the cell viability or proliferation was much smaller than in the case of light-pigmented melanocytes (HEMn-LP) [25].

The mitogen-activated protein kinase (MAPK) pathway, also known as the RAS-RAF-MEK-ERK signaling cascade, transmits cellular signals to effectors to regulate physiological processes such as cell proliferation, differentiation, viability, and death. High expression and activity of p44/p42 MAPKs promote cell survival and proliferation and can lead to inhibition of apoptosis. Low expression has the opposite effect [33,34,35].

**Figure 3:**
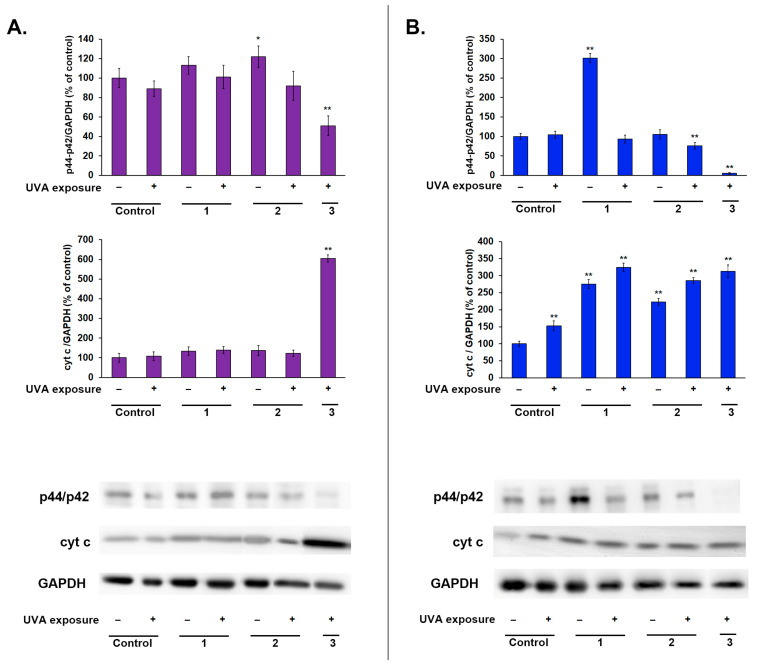
Western blot analysis of p44/p42 MAPK and cytochrome c proteins in melanocytes (**A**) and fibroblasts (**B**) exposed to the drug and UVA in three treatment schemes. Corresponding representative blot images are presented; * *p* < 0.05, ** *p* < 0.001 vs. control.

The results of the Western blot analysis revealed that in melanocytes, the downregulation of p44/p42 MAPK occurred only in Treatment Scheme 3—Figure 3A. In fibroblasts, this experimental treatment was also found to reduce the level of this protein and, furthermore, also in cells exposed to UVA in Treatment Scheme 2—Figure 3B. In Treatment Scheme 1, we only observed a significant difference between the levels of the protein in UVA-exposed and unexposed cells, as ketoprofen alone significantly increased p44/42 MAPK expression; however, UVA irradiation notably reduced it. The largest decrease was found in the model where the drug was present in the PBS during irradiation (Treatment Scheme 3). These changes, once again, may indicate the antiproliferative effect of the drug when combined with UVA radiation.

### 2.2. The Level of Reduced Thiols in Melanocytes and Fibroblasts Exposed to the Drug and Radiation in the Three Experimental Schemes

The redox state of intra- and extracellular thiols plays a key role in the structure and function of the proteins, in the control of transcription factor activity, and in the antioxidant defense [36,37]. Thiol compounds are a component of the thiol–disulfide redox buffer, which counteract oxidative stress as they act as free radical scavengers and also metal ion chelators. In addition, thiols inhibit the oxidation of low-density lipoprotein (LDL) in human plasma [37,38,39]. One of these substances is glutathione (GSH), which is a substrate in several specific redox reactions and participates in the reduction of protein disulfide bridges, transforming into the oxidized form (GSSG). Low levels of reduced glutathione in the cell may indicate an imbalance in the cellular oxidation–reduction state [40,41,42].

**Figure 4:**
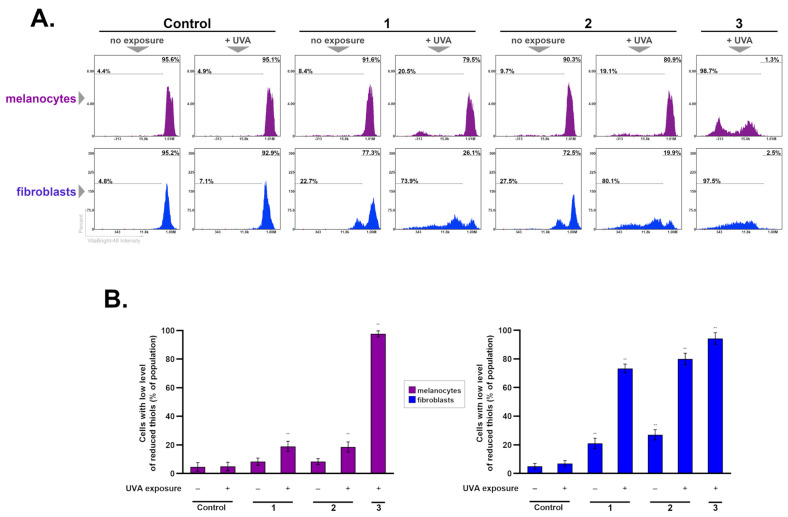
The analysis of reduced thiols in melanocytes and fibroblast after exposure to the drug and UVA—three treatment schemes (1–3). Histograms presenting the changes in thiol levels in cells exposed to ketoprofen in concentrations of 1.0 mM and UVA irradiation (**A**). Bar graph showing the thiol level of melanocytes and fibroblasts (**B**). The results are presented as the mean values ± SD. ** *p* < 0.001 vs. control.

To evaluate the impact of ketoprofen and irradiation with UVA on the level of GSH, cytometric analysis using a thiol group-specific fluorescent dye, VitaBright-48™ (VB48), was performed. The results revealed a change in the GSH oxidation status in both melanocytes and fibroblasts—Figure 4.

The obtained results showed that the exposure of melanocytes to only one of the factors, i.e., UVA radiation or the drug, did not decrease the cellular level of glutathione in its reduced form. However, the combination of ketoprofen with UVA caused a significant change in the intracellular thiol level. In melanocytes treated with experimental schemes 1 and 2 exposed to UVA, a statistically significant increase was noted when compared to control. The largest difference was found in the group of Treatment Scheme 3 (cells incubated with the drug during irradiation)—the percentage of cells with low vitality (exhibiting low levels of GSH) elevated by 95%, as compared with the controls.

In fibroblasts, an incubation with ketoprofen alone was found to elevate intracellular thiol levels; however, its effect was enhanced by UVA irradiation. In all of the tested groups irradiated with UVA, a large increase in the fraction of fibroblasts with low GSH levels was observed, that is, in Treatment Scheme 1, an increase of 68%, in Treatment Scheme 2, an increase of 75%, and in Treatment Scheme 3, an increase of 95%, when compared to the controls. These results, similar to the analysis of cell viability and proliferation, demonstrate that pre- and post-incubation with ketoprofen and UVA radiation (Schemes 1 and 2) have a stronger effect on fibroblasts than melanocytes.

In our previous studies, we showed that GSH disruption in melanoma cells as a result of exposure to various agents, such as lomefloxacin or ketoprofen with UVA irradiation, correlated well with the production of ROS, indicating that the loss of GSH may result from excessive oxidative stress [25,43]. Therefore, we may hypothesize that the changes in the cellular thiol levels discovered in the current study are related to the overproduction of ROS.

### 2.3. The Influence of the Treatment Schemes on Mitochondrial Transmembrane Potential

Mitochondria are organelles whose main function is to provide the energy necessary to carry out cellular processes. Mitochondria are involved in, among others, apoptosis, necrosis, and lipid oxidation [44]. Proper functioning of these organelles ensures the maintenance of normal transmembrane potential, which results from the impermeability of the mitochondrial membrane to cations. The consequence of this is a proton gradient on both sides of the membrane necessary for ATP synthesis [45,46].

During oxidative stress, the mitochondrial membrane can be permeabilized by peroxidation of lipids contained within it or by opening specific channels, which is regarded as the main mechanism. It leads to a decrease in mitochondrial membrane potential, a characteristic that is considered a marker of irreversible cell death [44,45,46,47].

Electrochemical potential (∆Ψm) on the inner mitochondrial membrane is one of the main parameters used to determine the condition of these organelles. Lowering ∆Ψm below a critical value promotes the opening of mitochondrial channels, which irreversibly leads to apoptosis. An increase in the permeability of mitochondria results in the release of apoptogenic proteins, such as cytochrome c, into the cytoplasm [47,48,49,50].

**Figure 5:**
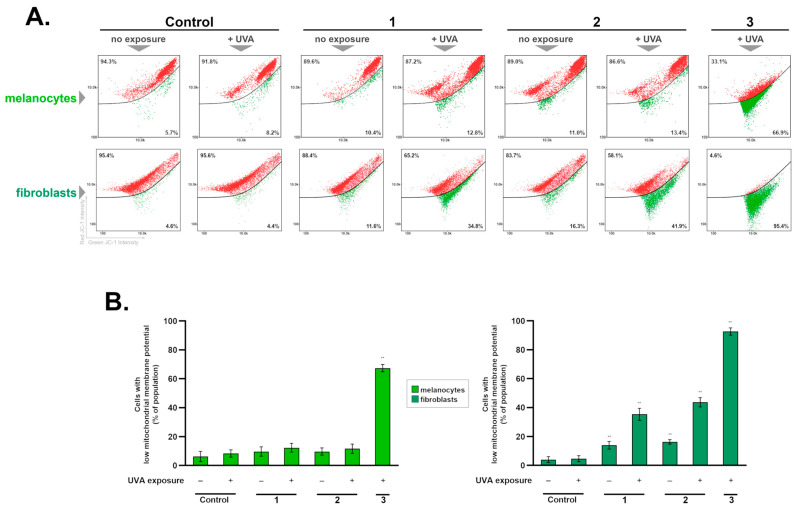
Combined exposure to ketoprofen (1.0 mM) and UVA radiation in the treatment schemes (1–3) cause changes in mitochondrial membrane potential in the melanocytes and fibroblasts. Data are shown as percentages of cells with permeabilized membranes (bottom part of the dot plot charts). The presented dot plot charts are representative of three independent experiments with similar results (**A**). Bar graph showing the percentages of depolarized cells after exposure to the three research models. Each bar graph represents mean ± SD from three independent experiments (**B**); ** *p* < 0.001 vs. controls.

To determine the optimal treatment scheme for testing the phototoxicity of ketoprofen, mitochondrial membrane potential was analyzed in normal skin cells (fibroblasts and melanocytes) exposed to the drug and radiation (UVA) in three different experimental regimens. The results confirmed again that fibroblasts are more prone to the tested factors, as a statistically significant decrease in subpopulations with low ∆Ψm was observed in groups exposed to ketoprofen alone. This effect was increased after exposure to UVA—there was an increase in the fraction of fibroblasts with a depolarized mitochondrial membrane in all of the treatment schemes, of about 30% (1), 45% (2), and 90% (3) when compared to the controls—Figure 5.

In melanocytes, subpopulations with decreased mitochondrial potential were found only in the group incubated with Treatment Scheme 3, where cells were exposed to the drug along with UVA irradiation. Similarly, previous studies also showed no effect of pre-incubation with ketoprofen before irradiation (UVA) on the mitochondrial potential of other pigmented cells, the HEMn-DP line. This is in line with the reported low sensitivity of melanin-containing cells to the first experimental scheme (pre-incubation with ketoprofen and UVA radiation) [25].

### 2.4. Changes in the Cell Cycle in Groups Incubated to the Tested Treatment Schemes

The cell cycle is one of the basic processes of a cell, which is a sequence of molecular events leading to the replication of genetic material and, subsequently, to division into two daughter cells. It is composed of four phases: G_1_, S, G_2_, and M, whose course is tightly regulated by complex mechanisms [51,52]. In addition, the sub-G_1_ phase can be distinguished, which is characterized by decreased DNA content, less than one equivalent of DNA of cells in the G_1_ phase, indicating DNA degradation as a result of the apoptosis process. The factor that may affect the cell cycle progression is oxidative stress, which can cause an arrest of the cycle in the G_1_, S, and/or G_2_ phases, leading to cell death [25,52].

**Figure 6:**
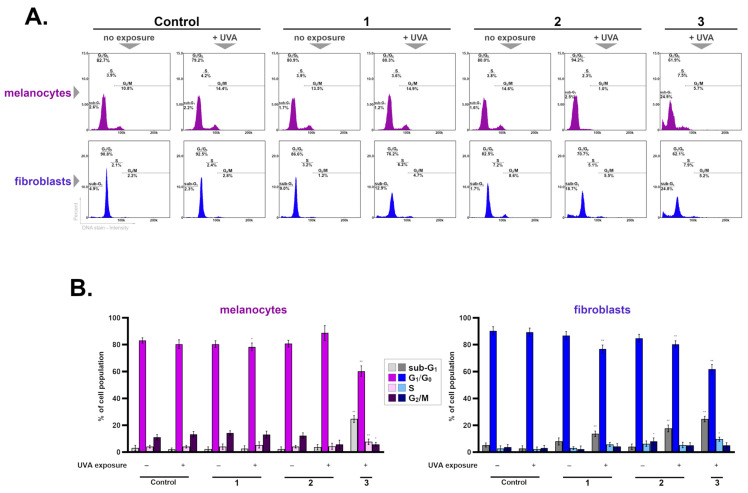
Effect of ketoprofen and UVA on cell cycle in melanocytes and fibroblasts. Representative histograms showing the distribution of cell cycle phases (**A**). Bar graphs representing mean values ± SD of three independent experiments performed in at least three repetitions (**B**); * *p* < 0.05, ** *p* < 0.001 vs. control.

Changes in the cell cycle of melanocytes were observed only in Treatment Scheme 3 (the drug combined with UVA radiation at the same time), where an increase in the fraction of sub-G_1_ and S phase cells was demonstrated. The largest difference was noted for the sub-G_1_ cell subpopulation, which was increased by approximately 25% when compared to the control. Moreover, in this experimental group, a reduction in the percentage of cells in the G_1_/G_0_ and G_2_/M phases was also found—Figure 6.

The analysis of fibroblasts revealed that in the cells exposed to ketoprofen and UVA in different experimental regimens, a significant elevation in the sub-G_1_ subpopulations was noted. For Treatment Schemes 1–3, they were estimated to be increased by 11%, 15%, and 25%, respectively, in comparison to controls. In the same groups, a decrease in the cells in the G_0_/G_1_ phase was observed—Figure 6.

The increase in the fraction of cells in the sub-G_1_ phase in skin cancer cells (melanoma) was also found after being exposed to ketoprofen and another drug with phototoxic potential, lomefloxacin, in combination with UVA irradiation [25,43]. The research results presented in this paper may suggest that ketoprofen in combination with UVA causes a disruption in DNA synthesis, which may explain the antiproliferative effect of the treatment schemes.

### 2.5. The Influence of Experimental Schemes on DNA Fragmentation in Melanocytes and Fibroblasts

A characteristic feature of the late stage of programmed cell death is DNA fragmentation. In the first stage of this process, large fragments of DNA are produced, reaching a size of 300 to 50,000 base pairs. Then, shorter fragments are made that have a length of multiples of about 200 nucleotides [53].

**Figure 7:**
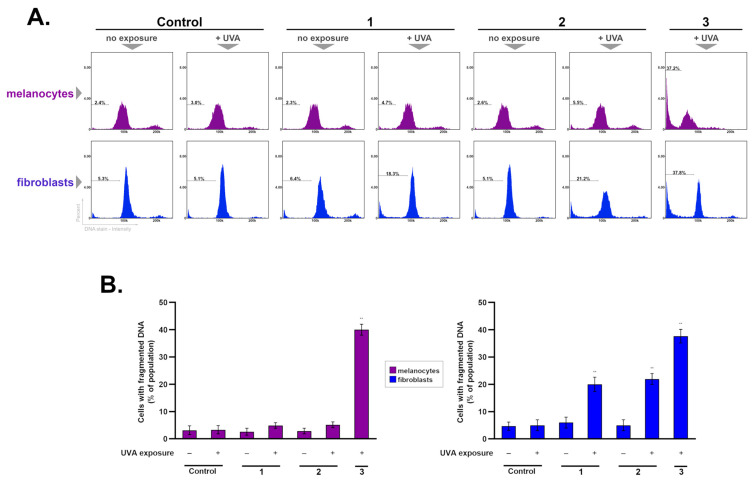
Effects of ketoprofen and radiation in three experimental schemes (1–3) on the DNA fragmentation process in melanocytes and fibroblasts. The presented histograms are representative of three independent experiments with similar results (**A**). Bar graphs show the percentages of cells with fragmented DNA—representing mean ± SD from three independent experiments (**B**); ** *p* < 0.001 vs. controls.

The induction of DNA fragmentation in fibroblasts and melanocytes after ketoprofen treatment and UVA irradiation in three experimental schemes was analyzed using a fluorescence image cytometer—Figure 7. The results showed that in fibroblasts incubated in all of the regimens where UVA was present, there was a high percentage of subpopulations with fragmented DNA. The strongest effect was observed in Treatment Scheme 3, where this fraction of cells was increased by over 30% compared to the control. In turn, the analysis conducted on melanocytes showed a statistically significant elevation of the percentage of cells with fragmented DNA only in Treatment Scheme 3, where it was increased by more than 35%, in comparison to the control.

In both melanocytes and fibroblasts, no notable effect on the percentage of cells with fragmented DNA was observed in Treatment Scheme 2 (pre-incubation and post-incubation with the drug) compared to Treatment Scheme 1 (cells only pre-incubated with the drug). This indicates that prolonged exposure to ketoprofen does not influence the phototoxic effect. Moreover, this suggests the lack of a cytotoxic effect of the drug.

One of the reasons for the induction of DNA fragmentation in the cell may be high levels of ROS [54]. They generate the formation of numerous oxidative damages in DNA, including damage to single nucleotides, strand breaks, and adducts [54,55].

### 2.6. Assessment of the Apoptosis Process After Exposure to Ketoprofen and UVA

Cytochrome c (cyt c) is a highly conserved hemoprotein located in mitochondria [56,57]. It plays multiple roles essential for cell physiology, including electron transfer within the respiratory chain between complexes III and IV [56]. Cyt c also acts as a ROS scavenger that neutralizes H_2_O_2_ and superoxide [58]. The function of this protein is also crucial for the intrinsic pathway of apoptosis—when the process is initiated, cyt c is released from mitochondria to the cytosol to bind with apoptotic protease-activating factor 1 (Apaf-1), which together with procaspase-9 form an apoptosome [56,58].

In order to evaluate the expression of cyt c in three experimental models, a Western blot analysis was conducted. The results showed that a statistically significant increase in cyt c level, when compared to control, occurred in all models performed on fibroblasts—Figure 3A. In fibroblasts, the strongest effect was observed in the groups exposed to the drug and UVA in all three models. However, in cells not exposed to UVA in Treatment Schemes 1 and 2, the increase in cyt c expression was also significantly elevated. In melanocytes, the significant intensification of cyt c expression was found only in cells incubated with Treatment Scheme 3, and it was almost 6-fold—Figure 3B.

The overexpression of mitochondrial respiratory chain proteins, including cyt c, is presumed to be an event that precedes cell death [59,60,61]. The results of the assessment of cyt c expression coincide to a large extent with the results of DNA fragmentation analysis. This suggests that the upregulation of this protein caused by the applied experimental schemes might be connected to cell demise. This might also be supported by the fact that the increase in cyt c expression took place in similar groups where the cell subpopulation with disrupted mitochondrial membrane potential and low levels of reduced thiols were detected. Interestingly, UVA exposure caused a noticeable elevation in cyt c levels in fibroblasts in each model, but not in melanocytes.

In normal cells, the activation of the p53 protein is a response to stress factors, including significant DNA damage. Depending on its degree, the p53 protein stops or slows down the cell cycle, which allows the repair of the genetic material and prevents replication of the errors in DNA. When the damage is too severe, the p53 protein directs the cell into the path of apoptosis. Therefore, the protein is involved in the regulation of proliferation, programmed death, and repair of genetic material. It is also involved in the control of processes related to metabolism cellular metabolism, autophagy, and cellular aging [62,63].

**Figure 8:**
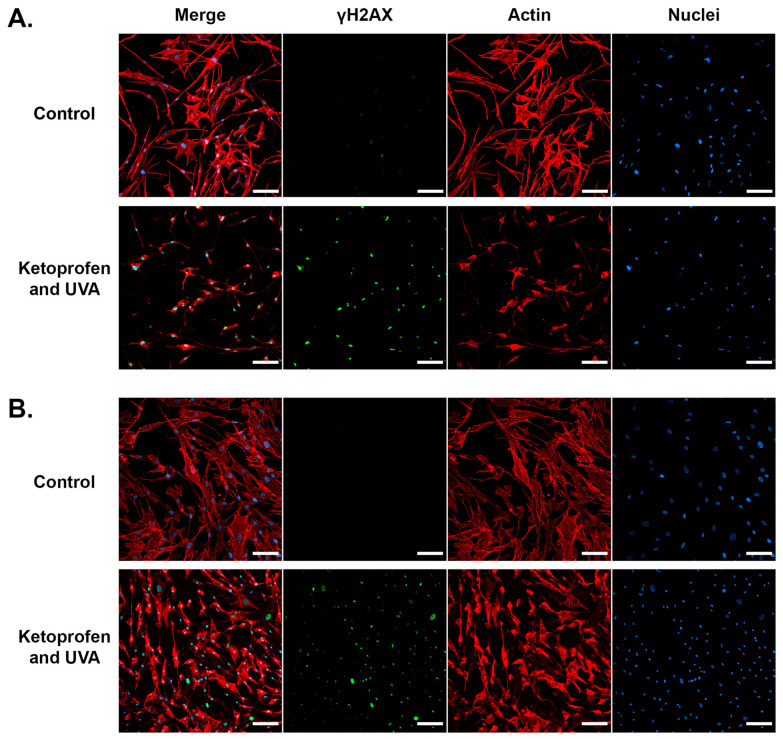
Confocal visualization of γH2AX. Representative confocal images of melanocytes (**A**) and fibroblasts (**B**) treated with ketoprofen and UVA radiation according to Treatment Scheme 3. The γH2AX, actin, and nuclei are visualized in green, red, and blue channels, respectively, as the signal from Alexa Fluor 488, Atto 565, and SYTO Deep Red Nucleic Acid Stain. Scale bar 100 µm.

**Figure 9:**
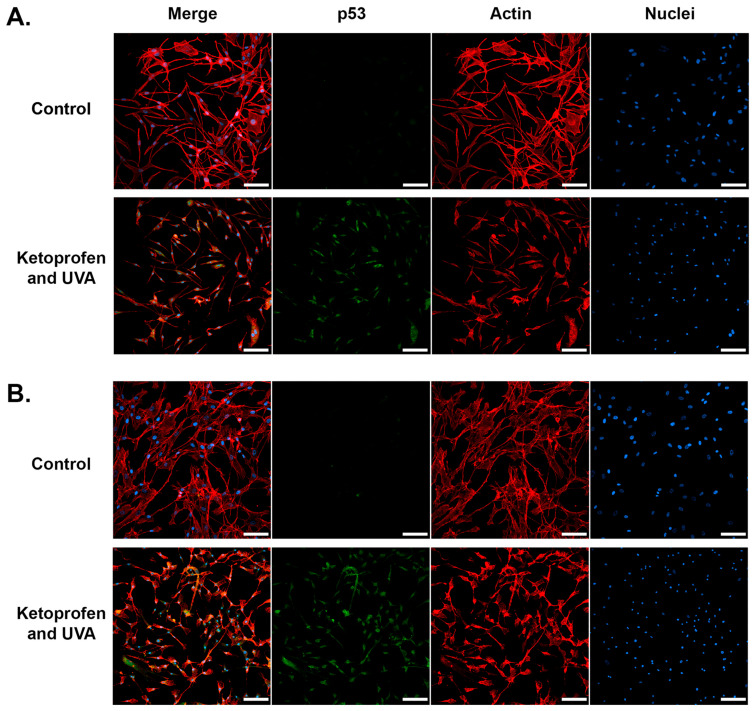
Confocal visualization of p53. Representative confocal images of melanocytes (**A**) and fibroblasts (**B**) treated with ketoprofen and UVA radiation according to Treatment Scheme 3. The p53, actin, and nuclei are visualized in green, red, and blue channels, respectively, as the signal from Alexa Fluor 488, Atto 565, and SYTO Deep Red Nucleic Acid Stain. Scale bar 100 µm.

Immunocytochemical analysis was performed to confirm DNA damage in the cells incubated with Treatment Scheme 3. The confocal analysis indicated that ketoprofen and UVA radiation increased the level of γH2AX (Figure 8) and p53 (Figure 9) in both melanocytes and fibroblasts. The phosphorylation of histone H2AX resulting in the formation of γH2AX is a very early event in the induction of DNA double-strand breaks [64]. Another well-known marker of cellular DNA damage is p53. Moreover, the microscopic observations (Figure 8 and Figure 9) revealed the significant cell shrinkage caused by the treatment, which may indicate apoptosis of both melanocytes and fibroblasts in these experimental groups.

## 3. Materials and Methods

### 3.1. Chemicals and Reagents

M-254 growth medium and a human melanocyte growth supplement-2 (HMGS-2) were produced by Cascade Biologics (Portland, OR, USA). Fibroblast Growth Medium, ketoprofen, amphotericin B solution (250 µg/mL), dimethyl sulphoxide, penicillin, phosphate-buffered saline (PBS), RIPA Buffer, PVDF membranes, and Phalloidin–Atto 565 were purchased from Sigma Aldrich Inc. (Taufkirchen, Germany). Neomycin sulfate was acquired from Amara (Kraków, Poland). Trypsin/EDTA was obtained from Cytogen (Zgierz, Poland). Via-1-Cassettes™ (acridine orange and DAPI fluorophores), NC-slides A8, as well as Solution 3 (1 µg/mL DAPI, 0.1% triton X-100 in PBS), Solution 5 (VB-48™ PI AO), Solution 7 (200 µg/mL JC-1), and Solution 8 (1 µg/mL DAPI in PBS), were obtained from ChemoMetec (Lillerød, Denmark). Primary rabbit monoclonal antibodies, anti-GAPDH, anti-p44/p42, anti-cytochrome c, and anti-γH2AX monoclonal antibody (Ser139, 20E3) were obtained from Cell Signaling (Danvers, MA, USA). Anti-p35 monoclonal antibody was acquired from Santa Cruz Biotechnology Inc. (Dallas, TX, USA). A Pierce BCA Protein Assay Kit, ECL Western Blotting Substrate, Alexa Fluor 488-conjugated anti-mouse antibody and Alexa Fluor 488-conjugated anti-rabbit antibody, and SYTO Deep Red Nucleic Acid Stain were obtained from Thermo Fisher Scientific (Waltham, MA, USA).

### 3.2. Cell Culture

Studies were performed on human light-pigmented epidermal melanocytes (HEMn-LP) and human dermal fibroblasts (HDFs), which were purchased from Cascade Biologics (Portland, OR, USA). All cells used in this research were from passages 4 to 9. The HEMn-LP cells were cultured in the growth medium M-254, which was supplemented with HMGS-2 and antibiotics: penicillin (100 µg/mL), amphotericin B (0.25 µg/mL), and neomycin sulfate (10 µg/mL). HDF cells were cultured in Fibroblast Growth Medium.

### 3.3. Treatment Schemes

Cells were seeded on culturing dishes and incubated in appropriate media for 24 h, and then 3 treatment schemes were established:

Treatment Scheme 1—The culture medium was replaced with a 1.0 mM ketoprofen solution. In parallel, control samples containing basal culture medium were prepared. After a 24-h incubation with ketoprofen solutions, the cells were exposed to UVA radiation. Immediately before irradiation, the drug solution and culture medium were replaced with PBS solution. During irradiation, the non-UVA-treated cells were placed in a CO_2_ incubator. After the irradiation process was completed, the PBS was replaced with the primary culture medium, and the cells were incubated consecutively for 24 h with the culture medium—Figure 1.

Treatment Scheme 2—The culture medium was replaced with 1.0 mM ketoprofen solution. In parallel, control samples containing basal culture medium were prepared. After a 24-h incubation with ketoprofen solutions, the cells were exposed to UVA light. Immediately before irradiation, the drug solution and culture medium were replaced with PBS solution. During irradiation, the non-UVA-treated cells were placed in a CO_2_ incubator. After the irradiation process was completed, the PBS was replaced with 1.0 mM ketoprofen solution, for another 24 h—Figure 1.

Treatment Scheme 3—Cells were cultured in the medium until irradiation. Immediately before irradiation, a solution of ketoprofen (1.0 mM) in PBS was added to the cells, then the cells were irradiated. After the irradiation process was completed, the drug dissolved in PBS was replaced with the culture medium, and the cells were incubated in the medium for another 24 h—Figure 1.

### 3.4. UVA Exposure

UVA irradiation was performed using a filtered lamp BVL-8.LM (Vilber Lourmat, France). The normal human cells were irradiated for 46 min at an intensity of 720 μW/cm^2^ (at dose 2 J/cm^2^).

### 3.5. Cell Viability

In order to investigate the effect of simultaneous treatment with ketoprofen and UVA radiation on viability, normal cells were seeded in Petri dishes at 1 × 10^6^ cells per plate in a dedicated medium. Drug and radiation treatments were performed according to previously described experimental models. The cells were suspended in the growth medium and loaded into Via1-Cassettes (ChemoMetec) containing the dyes. Next, cell viability was assessed using a NucleoCounter NC-3000 fluorescence cytometer (ChemoMetec). The method is based on the staining of cells (non-fixed) with DAPI—detection of dead cells. The parameter was calculated based on the formula:

(1) $$\%\mathrm{Viability}=\frac{\mathrm{The}\;\mathrm{total}\;\mathrm{concentration}\;\mathrm{of}\;\mathrm{cells}-\mathrm{The}\;\mathrm{concentration}\mathrm{of}\;\mathrm{non-viable}\;\mathrm{cells}}{\mathrm{The}\;\mathrm{total}\;\mathrm{concentration}\;\mathrm{of}\;\mathrm{cells}}\times 100\%$$

### 3.6. Cell Proliferation

The assessment of the proliferation of normal human cells was made using the NucleoCounter^®^ NC-3000™ (ChemoMetec, Lillerød, Denmark). The results are presented as the ratio of the number of cells in the test samples relative to the control. Ketoprofen and irradiation treatments were performed according to previously described experimental models. Next, the sample of cell suspension was loaded into the Via1-Cassette™ and stained with fluorescent dyes contained in the cassette: acridine orange (stains all cells) and DAPI (stains cells with damaged cell membrane). The analysis was performed according to the “Cell Viability and Cell Count Assays” protocol of the cytometer-controlling software (NucleoView NC-3000 software). The proliferation ratio was expressed as:

(2) $$\mathrm{Proliferation}\;\mathrm{ratio}=\frac{\mathrm{The}\;\mathrm{total}\;\mathrm{amount}\;\mathrm{of}\;\mathrm{cells}\;\mathrm{in}\;\mathrm{sample}}{\mathrm{The}\;\mathrm{total}\;\mathrm{amount}\;\mathrm{of}\;\mathrm{cells}\;\mathrm{in}\;\mathrm{control}}$$

### 3.7. Western Blotting Analysis

After treatment, cells were lysed using RIPA buffer containing phosphatase and protease inhibitors. Prepared lysates were centrifuged and stored (at −86 °C) until the assessment of total protein concentration (Pierce™BCA Protein Assay Kit—Waltham, MA, USA), according to the producer’s protocol, and Western blotting analysis. Protein extracts (20 µg/lane) were separated on an SDS (10%)–polyacrylamide gel electrophoresis and transferred to PVDF membranes (Sigma-Aldrich Inc., Taufkirchen, Germany). The membranes were incubated for 1 h in blocking buffer (a solution of 5% non-fat milk and Tris-buffered saline with Tween 20) and washed. The proteins were detected using the primary monoclonal antibodies rabbit anti-GAPDH (1:1000), rabbit anti-p44/42 (1:1000), and rabbit anti-cytochrome c (1:1000) diluted in blocking buffer. Next, the membranes were washed with Tris-buffered saline with Tween 20 and incubated for 1.5 h (at room temperature) with appropriate secondary antibody, diluted previously in blocking buffer (1:10,000). In the final step, the protein signals were detected using ECL western reagent (Waltham, MA, USA). The studies were made using a G: Box Chemi-XT4 Imaging System and GeneTools Software 4.0 (Syngene, Cambridge, UK). The results were normalized using the level of GAPDH and expressed as the percentage of control.

### 3.8. Assessment of Intracellular GSH Level

Cells (0.5 × 10^6^) incubated in the treatment schemes were resuspended with 190 µL of PBS in an Eppendorf-type tube and 10 μL of Solution 5 (a specific dye—VitaBright-48™) was added. The stained cells were loaded into the NC-Slide A8 and measured using the “Vitality Assay” protocol in the NucleoCounter^®^ NC-3000™ fluorescent imaging cytometer (Denmark). The obtained histograms were used to demarcate the percentage of cells with low cellular GSH levels and cells with high cellular GSH levels (healthy cells).

### 3.9. Mitochondrial Membrane Potential

Cells of 1 × 10^6^ were resuspended with 1 mL of PBS in an Eppendorf-type tube and 12.5 μL of Solution 7 (200 µg/mL JC-1) was added and incubated for 30 min at 37 °C. Then, the samples were centrifuged, the supernatant was removed, and the cell pellet was washed twice with PBS. Cells were resuspended in 0.25 mL of Solution 8 (1 µg/mL DAPI in PBS) and immediately analyzed on NC-Slide A8 two-chamber slides using the “Mitochondrial Potential Assay” protocol. The results presented in the form of scatterplots were used to demarcate the percentage of polarized/healthy cells and depolarized/apoptotic cells. Mitochondrial depolarization was exhibited as a decrease in the red/green fluorescence intensity ratio.

### 3.10. DNA Fragmentation Assay

Cells of 1 × 10^6^ obtained from the incubations in the treatment schemes described earlier were resuspended in 0.5 mL of PBS in Falcon tubes and 4.5 mL of cold 70% ethanol was added. The prepared cell suspensions were stored at 4 °C for at least 12 h. The samples were then centrifuged, and the supernatant was removed. The remaining cell pellets were resuspended in 1 mL of PBS and centrifuged again. After removing the supernatant, the resulting cell pellets were resuspended in 0.5 mL of Solution 3 (1 μg/mL DAPI, 0.1% Trixton X-100 in PBS) and incubated for 5 min at 37 °C. The stained cells were analyzed with a fluorescence image cytometer using the “DNA Fragmentation Assay” protocol. The obtained histograms were used to demarcate the subpopulation of the tested cell culture with fragmented DNA.

### 3.11. Cell Cycle Analysis

Cells from each of the experimental groups, after incubation under the conditions described above, in the amount of 1 × 10^6^ cells/sample were resuspended in 0.5 mL of PBS, and then 4.5 mL of cold 70% ethanol was added and placed on ice at 4 °C for at least 48 h. Next, the cells were centrifuged, the supernatant was removed, resuspended in 1 mL of PBS, and centrifuged again. After the washing step, 0.5 mL of Solution 3 (1 µg/mL DAPI, 0.1% triton X-100 in PBS) was added to the remaining cell pellets and incubated for 5 min. The stained cells were analyzed by cytometric techniques using the “Fixed Cell Cycle-DAPI” protocol. The results were presented in DNA content histograms, where different phases of the cell cycle were demarcated.

### 3.12. Immunocytochemistry

Melanocytes and fibroblasts were cultured on sterile coverslips in the recommended growth media. Then, the cells were exposed to ketoprofen and/or UVA radiation according to the description concerning Treatment Scheme 3. After 24 h of incubation, the samples were fixed and permeabilized with 4% paraformaldehyde and 0.1% Triton X-100. After blocking the reaction with 0.25% glycine and 3% bovine serum albumin (BSA), the cells were incubated overnight at 4 °C with an anti-γH2AX monoclonal antibody (Ser139, 20E3) (1:200) or anti-p53 monoclonal antibody (1:200) with 3% BSA. After three rinses in PBS, the cells were incubated for 2 h at room temperature with Alexa Fluor 488-conjugated secondary antibodies (1:200). Phalloidin–Atto 565 and SYTO Deep Red Nucleic Acid Stain were used to label the tested protein (γH2AX or p53), actin, and nuclei. The dyes were applied in the concentrations recommended by the manufacturer. Immunofluorescent images were visualized using a confocal microscope Nikon Eclipse Ti-E A1R-Si and Nikon NIS Elements AR software 4.51 (Nikon Instruments, Amsterdam, The Netherlands).

### 3.13. Statistical Analysis

Statistical analysis of the results was performed using GraphPad Prism 8.2.0 (GraphPad Software, San Diego, CA, USA). In all experiments, the means and standard deviations (SDs) of at least three separate experiments conducted in triplicate were calculated. Statistical significance was determined by one-way ANOVA and two-way ANOVA, as well as Dunnett’s and Tukey’s multiple comparison tests. In all cases, the statistical significance was found for the *p*-value to be lower than 0.05.

## 4. Conclusions

Phototoxicity is one of the most common skin adverse reactions related to pharmacotherapy. In the case of ketoprofen, our study showed that it exhibits the strongest phototoxic potential when it was present in the PBS solution during UVA irradiation. This treatment scheme was found to be effective against both pigmented and non-pigmented cells of the skin, which were melanocytes and fibroblasts, respectively. In addition, ketoprofen in combination with UVA radiation induced apoptotic cell death, manifested by disruption of thiol levels, decreased mitochondrial membrane potential, cell cycle disruption, DNA fragmentation, and activation of proteins related to cell death, such as p53 and cytochrome c. These results indicate that the highest risk of photosensitivity reactions occurs after direct contact with the drug and UV irradiation. These findings may help avoid adverse effects of ketoprofen related to the phototoxicity of the drug and develop guidelines for the safe use of the drug.

## Data Availability

The data that support the findings of this study are available from the corresponding author upon reasonable request.
